# Assessment of breast cancer awareness among female pharmacy students at a university in Turkey

**DOI:** 10.1186/s12909-024-05353-x

**Published:** 2024-04-03

**Authors:** Aslınur Albayrak, Kayhan Nuri Cengiz

**Affiliations:** 1 Department of Clinical Pharmacy, Faculty of Pharmacy, Suleyman Demirel University, Isparta, Turkey; 2 Department of Clinical Pharmacy, Institute of Health Sciences, Marmara University, Istanbul, Turkey

**Keywords:** Awareness, Breast cancer, Knowledge, Students, University, Turkey

## Abstract

**Background:**

Female breast cancer is the most frequently diagnosed cancer, and knowledge of breast cancer risk factors, and symptoms is crucial for early diagnosis and prevention. This study aims to evaluate breast cancer awareness among female students at a pharmacy faculty in Turkey.

**Methods:**

A cross-sectional online survey study was conducted among female students at the Suleyman Demirel University Faculty of Pharmacy between 2 November and 17 November 2023, in Isparta, Turkey.

**Results:**

This survey was answered by 237 (74.5%) female students. The median breast cancer risk factors score was 8 (IQR, 5–11), and the median breast cancer symptoms score was 5 (IQR, 2–8). Additionally, the breast cancer risk factors score was 46.16% (mean/max = 8.31/18, SD = 4.33) and the breast cancer symptom score was 58.5% (mean/max = 4.68/8, SD = 2.8). Few of the respondents (26.2%, and 20.3%, respectively) knew breast cancer risk factors such as late menopause, and no childbirth experience. Most respondents correctly answered symptoms of breast cancer, such as a painless and palpable breast lump, indrawing of the nipple, and sudden changes in breast shape (76.8%,44.3%, and 67.1% respectively). The students’ sources of information were medical websites (29.5%), social media (27%), physicians (22.8%), friends & family (14.8%), and pharmacists (5.9%).

**Conclusions:**

This study showed that students’ knowledge of breast cancer risk factors was poor, but their knowledge of breast cancer symptoms was acceptable. Breast cancer awareness courses should be included in faculties. Additionally, more attention should be given to different educational interventions such as social media, television, and brochure distribution.

## Background

Female breast cancer is the most frequently diagnosed cancer, with an estimated 2.3 million new cases [1]. Death from breast cancer is higher in low- and middle-income countries [2]. Additionally, the incidence of breast cancer in Turkey is gradually increasing. It increased to 43.8% in 2015 and 48.6% in 2018 [3].

Increasing age, early menarche, late menopause, using oral contraceptives for more than 5 years, receiving hormone therapy after menopause, exposure to radiation in childhood or adolescence, late pregnancy, low parity, never having given birth, lack of breastfeeding, family history of breast cancer are among the factors that increase the risk of breast cancer [4–6]. Some lifestyle risk factors also increase the risk of breast cancer. These include low physical activity, being overweight or obese, stress, smoking and alcohol consumption, high consumption of red meat, low consumption of vegetables and fruits, and high consumption of fatty foods [7, 8].

Early diagnosis of breast cancer is important in the effective treatment of the disease and increasing survival [9]. Breast self-examination (BSE), clinical breast examination (CBE), and mammography are the most commonly used screening techniques. BSE is an inexpensive, simple screening method that relies on the person detecting any changes or abnormalities in their breasts [9, 10]. Early diagnosis and prevention need to have adequate knowledge about breast cancer risk factors, signs, and symptoms, and it is the responsibility of healthcare providers to educate women about this [11].

In studies conducted in developing countries, students’ knowledge levels about breast cancer and BSE were generally found to be insufficient [12–17]. In a study conducted in Saudi Arabia, only 4.2% of students had sufficient knowledge about BSEs [12]. A study in Pakistan found that students did not have sufficient knowledge about breast cancer risk factors, symptoms, and BSE [13]. In a study conducted in Nigeria, pharmacy students’ breast cancer risk factors knowledge scores were 10.5% good, 60.1% average, 29.4% bad, and 42.1% of their BSE knowledge scores were good, 39.5.5% fair, 18.4% was poor [14]. In Syria, the average knowledge rate of students was (57.5%) and 70% of students did not know about mammography. However, most of the students (86.7%) had good knowledge about BSE [15]. Two studies conducted in the United States also found the level of knowledge about breast cancer among university students to be insufficient [16, 17].

Although the results of studies conducted in Turkey varied, there was generally a lack of awareness about breast cancer and BSE practice [18–21]. In the study conducted by Türkmen et al. [18] 34.6% of the students knew how BSE should be performed, and 14.3% knew when it should be performed.14.1% of the students were doing BSE once a month. In the study conducted by Koc et al. [19] a total of 73.3% of the students had heard of BSE. Only half of these students had acquired additional information about BSE. Half of the students had performed BSE and 33.3% had performed BSE regularly. In a study conducted on medical faculty students, 66.8% of the students stated that they did not perform BSE, and 68.8% stated that not having sufficient knowledge was the reason for this [20]. In a different study conducted on medical faculty students, it was determined that 69.6% of the students knew how to do BSE and 42.9% of them did BSE [21].

As health consultants, pharmacists make positive contributions to the awareness of patients and their relatives about cancer diagnosis and treatment [22, 23]. Studies evaluating the knowledge and awareness of pharmacy faculty students, as future pharmacists, about cancer, are important in terms of revealing the lack of knowledge on this subject. Therefore, this study aims to evaluate breast cancer awareness among female students at a pharmacy faculty in Turkey.

## Methods

### Study design

A cross-sectional online survey study was conducted among female students at the Suleyman Demirel Faculty of Pharmacy between 2 November and 17 November 2023, in Isparta, Turkey. Ethics committee permission was obtained from the Suleyman Demirel University Clinical Research Ethics Committee (No: 194 / Date:31.10.2023).

### Inclusion and exclusion criteria

Only female students over the age of 18 who were studying at the undergraduate level at Suleyman Demirel University Faculty of Pharmacy and agreed to participate were included in the study. Male students and graduate students (master’s, Ph.D.) were not included.

### Sampling

Using the Raosoft sample size calculator, the sample size was found to be a minimum of 175 female students with a 5% margin of error, a 95% confidence interval, and a 50% response rate. x = Z(^c^/100)^2^r(100-r), n = ^Nx^/((N-1)E^2^ + x), E = Sqrt[^(N−n)x/^n(N-1)]. N is the population size, r is the proportion of responses of interest, and Z(c/100) c is the critical value for the confidence level [24]. A convenience sampling method was used as the sampling method.

### Study instrument and data collection

This survey was created by revising the previously published study [25]. Permission was obtained from the corresponding author of the article. Expert opinions were obtained from 2 pharmacists and 1 doctor who are experts in their fields. They evaluated the questions in the survey in terms of content and scope. Minor revisions were made to the questions. In order to examine the questions in terms of scope and clarity, a pilot study was conducted with a total of 30 students from each grade. Since the questions were understandable, no revisions were made and the final version was created. Cronbach’s alpha score was calculated as 0.832 for risk factors and 0.896 for symptoms, indicating good internal consistency.

The survey was created with Google Form and distributed to students through class groups via the Whatsapp application before lessons. It took students approximately 10–15 min to answer the survey. On the first page of the survey, it was announced that the study would be conducted for scientific purposes and that the data would be kept confidential. Those who chose “I have read and approved” participated in the survey. In order to prevent participants from giving duplicate answers, the “Limit 1 answer” setting was applied in Google Form.

The survey consisted of 32 questions. The first 5 questions were about sociodemographic characteristics, 18 questions were about breast cancer risk factors, 8 questions were about breast cancer symptoms, and 1 question was about the source of breast cancer information.

### Statistical analysis

Statistical Package for Social Sciences (SPSS) 20.0 was used to analyze the data. Variables were described as mean-standart deviation (SD), median-interquartile range (IQR), number, and percentage. The normality of the data was determined using the Kolmogorov-Smirnov test. When two groups were compared, Student’s T-test was used when the variables were normally distributed and Mann-Whitney U was used when they were not normally distributed. The Chi-square test was used to compare categorical variables. *P* value < 0.05 was considered statistically significant.

In the knowledge section, correct answers were scored as 1 point and incorrect answers were scored as 0 point. ≥50% of the total score was considered good knowledge. A score of ≥ 9 for breast cancer risk factors was associated with a good level of knowledge, and a score of ≥ 4 for breast cancer symptoms was associated with a good level of knowledge.

## Results

There were a total of 318 female students in the faculty, the survey was answered by 237 (74.5%) female students. The median age of respondents was 21 (IQR,20–22). Most respondents were not regular smokers or alcohol users (88.2% and 94.1%, respectively). Table 1 shows the socio-demographic characteristics of the students.

**Table 1:** Demographic characteristics of students

Variables	n (%)
**Age median (IQR)**	21 (20–22)
**Year of study**	
First year	53 (22.4)
Second year	47 (19.8)
Third year	50 (21.1)
Fourth year	36 (15.2)
Fifth year	51 (21.5)
**Regular smoking**	
Yes	28 (11.8)
No	209 (88.2)
**Regular alcohol use**	
Yes	14 (5.9)
No	223 (94.1)
**Mother’s education level**	
Illiterate	9 (3.8)
Elementary school	60 (25.3)
Secondary school	27 (11.4)
High school	71 (30)
University	65 (27.4)
Postgraduate	5 (2.1)

IQR: Interquartile range

The median breast cancer risk factors score was 8 (IQR, 5–11), and the median breast cancer symptoms score was 5 (IQR, 2–8). Additionally, the breast cancer risk factors score was 46.16% (mean/max = 8.31/18, SD = 4.33) and the breast cancer symptom score was 58.5% (mean/max = 4.68/8, SD = 2.8). In total, 44.7% had good knowledge about risk factors and 71.3% had good knowledge about symptoms. Most respondents (62%) knew that a history of breast cancer in a first-degree relative was a risk factor for breast cancer. Students (38.8%, 37.6%, 48.1%, and 68.4%, respectively) stated that using oral contraceptive pills for more than 5 years, postmenopausal hormone therapy, history of benign breast disease, and radiation therapy during childhood or adolescence are risk factors for breast cancer. Few of the respondents (19.8%, 26.2%, 21.1%, 20.3%, and 34.6% respectively) knew breast cancer risk factors such as menstruation before the age of 12, late menopause, giving birth for the first time after the age of 30, no childbirth experience, and lack of breastfeeding. Risk factors such as being overweight and obese, being over 40 years of age, and past or present smoking or alcohol consumption were answered correctly by 58.2%, 50.6%, and 79.3% of respondents, respectively. Table 2 shows students’ knowledge about breast cancer risk factors. Stress was the risk factor answered most correctly (93.2%) by respondents [7, 26]. In addition, 22.4%, 45.6% and 55.3% of the students, respectively, declared high consumption of red meat, low consumption of vegetables and fruits and high consumption of fatty foods as breast cancer risk factors.

**Table 2:** Students’ knowledge about breast cancer risk factors

Questions	Yes (N,%)	No (N,%)	Don’t know (N,%)
History of breast cancer in a first-degree relative	147 (62)	62 (26.2)	28 (11.8)
Using oral contraceptive pills for more than 5 years	92 (38.8)	60 (25.3)	85 (35.9)
Hormone therapy after menopause	89 (37.6)	52 (21.9)	96 (40.5)
History of benign breast disease	114 (48.1)	49 (20.7)	74 (31.2)
High radiation to the chest or breast during childhood or adolescence (radiation therapy)	162 (68.4)	38 (16)	37 (15.6)
Menstruation before age 12	47 (19.8)	76 (32.1)	114 (48.1)
Late menopause (after age 55)	62 (26.2)	58 (24.5)	117 (49.4)
Giving birth for the first time after age 30	50 (21.1)	79 (33.3)	108 (45.6)
Not having given birth	48 (20.3)	85 (35.9)	104 (43.9)
Low physical activity	119 (50.2)	45 (19)	73 (30.8)
Being overweight and obese	138 (58.2)	41 (17.3)	58 (24.5)
Being over 40 years old	120 (50.6)	55 (23.2)	62 (26.2)
Lack of breastfeeding	82 (34.6)	55 (23.2)	100 (42.2)
Smoking or alcohol consumption in the past or present	188 (79.3)	19 (8)	30 (12.7)
Stress	221 (93.2)	1 (0.4)	15 (6.3)
High consumption of red meat	53 (22.4)	60 (25.3)	124 (52.3)
Low consumption of vegetables and fruits	108 (45.6)	42 (17.7)	87 (36.7)
High consumption of fatty foods	131 (55.3)	33 (13.9)	73 (30.8)

Most respondents correctly answered symptoms of breast cancer, such as a painless and palpable breast lump, painless mass under armpit, bleeding or discharge from the nipple, indrawing of the nipple, wound around the nipple, redness of the breast skin and abrupt changes in breast size and shape (76.8%, 67.1%, 58.2%, 44.3%, 44.7%, 42.6% 67.9% and 67.1% respectively). Table 3 shows students’ knowledge about breast cancer symptoms.

**Table 3:** Students’ knowledge about breast cancer symptoms

Questions	Yes (N,%)	No (N,%)	Don’t know (N,%)
Painless and palpable breast lump	182 (76.8)	34 (14.3)	21 (8.9)
Painless mass under armpit	159 (67.1)	38 (16)	40 (16.9)
Bleeding or discharge from the nipple	138 (58.2)	35 (14.8)	64 (27)
Nipple pulling inwards	105 (44.3)	40 (16.9)	92 (38.8)
Wound around the nipple	106 (44.7)	38 (16)	93 (39.2)
Redness of the breast skin	101 (42.6)	39 (16.5)	97 (40.9)
Abrupt changes in breast size	161 (67.9)	31 (13.1)	45 (19)
Abrupt changes in breast shape	159 (67.1)	30 (12.7)	48 (20.3)

**Fig. 1:**
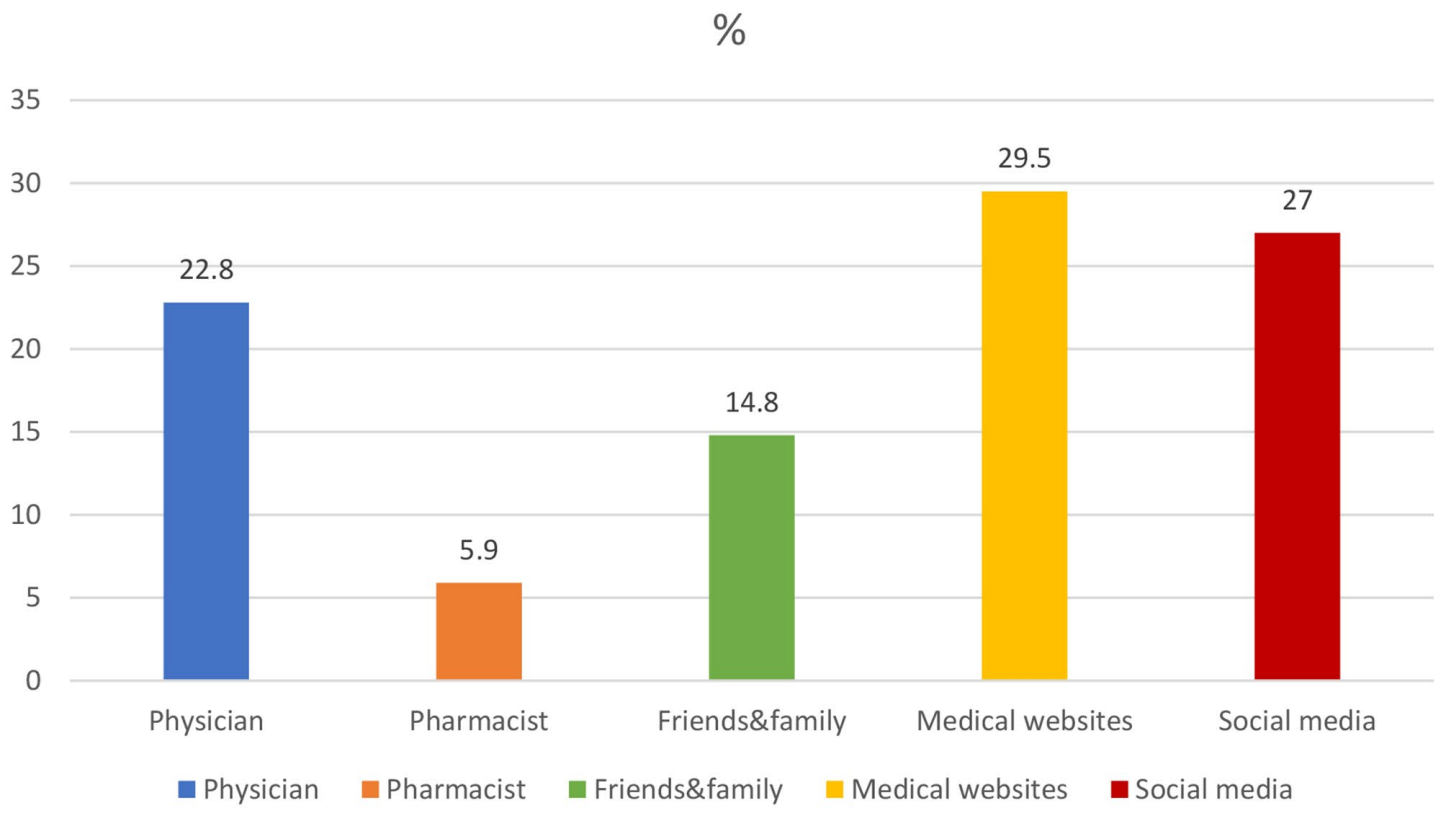
Students’ source of information about breast cancer

The students’ sources of information were medical websites (29.5%), social media (27%), physicians (22.8%), friends & family (14.8%) and pharmacists (5.9%) (Fig. 1).

There was no statistical relationship between students’ sociodemographic variables and their knowledge of breast cancer risks and symptoms (*p* > 0.05) (Table 4).

**Table 4:** Comparison of sociodemographic variables with the level of knowledge about breast cancer risks and symptoms

Variables	Breast cancer risks knowledge level			Breast cancer symptoms knowledge level		
	Poor (%) n	Good (%) n	*p*	Poor (%) n	Good (%) n	*p*
**Age, mean ± SD**	20.78 ± 1.66	20.99 ± 1.56	0.318	20.56 ± 1.7	21 ± 1.57	0.058
**Year of study**						
First year	30 (56.6)	23 (43.4)	0.127	18 (34)	35 (66)	0.338
Second year	22 (46.8)	25 (53.2)		13 (27.7)	34 (72.3)	
Third year	35 (70)	15 (30)		17 (34)	33 (66)	
Fourth year	20 (55.6)	16 (44.4)		11 (30.6)	25 (69.4)	
Fifth year	24 (47.1)	27 (52.9)		9 (17.6)	42 (82.4)	
**Regular smoking**						
Yes	18 (64.3)	10 (35.7)	0.413	11 (39.3)	17 (60.7)	0.273
No	113 (54.1)	96 (45.9)		57 (27.3)	152(72.7)	
**Regular alcohol use**						
Yes	7 (50)	7 (50)	0.895	4 (28.6)	10 (71.4)	1
No	124 (55.6)	99 (44.4)		64 (28.7)	159 (71.3)	
**Mother’s education level**						
Illiterate	5 (55.6)	4 (44.4)	0.846	3 (33.3)	6 (66.7)	0.2
Elementary school	33 (55)	27 (45)		15 (25)	45 (75)	
Secondary school	13 (48.1)	14 (51.9)		7 (25.9)	20 (74.1)	
High school	41 (57.7)	30 (42.3)		19 (26.8)	52 (73.2)	
University	35 (53.8)	30 (46.2)		20 (30.8)	45 (69.2)	
Postgraduate	4 (80)	1 (20)		4 (80)	1 (20)	
**Source of Information**						
Physician	32 (59.3)	22 (40.7)	0.951	13 (24.1)	41 (75.9)	0.349
Pharmacist	7 (50)	7 (50)		3 (21.4)	11 (78.6)	
Friends&family	20 (57.1)	15 (42.9)		15 (42.9)	20 (57.1)	
Medical websites	38 (54.3)	32 (45.7)		19 (27.1)	51 (72.9)	
Social media	34 (53.1)	30 (46.9)		18 (28.1)	46 (71.9)	

SD: Standard deviation

## Discussion

In our study, the knowledge level of female students studying at a pharmacy faculty in Turkey about breast cancer risk factors and symptoms was evaluated. There were limited studies on this subject among pharmacy students [14, 27]. According to our study, students’ knowledge of breast cancer risk factors was poor, but their knowledge of breast cancer symptoms was acceptable.

Similar to our study, in most of the studies conducted, students had poor and limited knowledge about breast cancer [25, 28–30]. It is very important to know the risk factors in the early diagnosis and prevention of breast cancer [31]. Having a history of breast cancer in a first-degree relative is an important risk factor for breast cancer [32]. In a study conducted in Ethiopia [29], 75.3% of students, in a study conducted in Syria [11], 92.4% of students, in a study conducted in Egypt [33], 57% of medical students and 31.5% of non-medical students knew that having a family history of breast cancer was a risk factor. In our study, the correct response rate (62%) was close to the previous studies.

In our study, very few students knew breast cancer risk factors such as menstruation before the age of 12, late menopause, and giving birth for the first time after the age of 30 (19.8%, 26.2%, 21.1% respectively). While the rates of correct answers to these questions were higher in studies conducted in Jordan and Ethiopia [28, 29], the studies conducted in Egypt and Pakistan [25, 33] were similar to our study. In a study conducted among medical faculty students in Pakistan [13], having children at an older age or not having children at all was known as a breast cancer risk factor in 28.9% of preclinical students and 48.6% of clinical students. Early menarche and late menopause were known as breast cancer risk factors by 14.8% and 23.4% of preclinical students and 20.3% and 29.7% of clinical students, respectively. In a study conducted in Egypt [33], medical students and non-medical students knew early menarche and late menopause as breast cancer risk factors at a rate of 34.2% and 21%, respectively. In a study conducted in the Midwestern USA [17], using oral contraceptives, and taking hormone replacement therapy were known as breast cancer risk factors by 14%, and 27% of the students, respectively. In a study conducted on medical faculty students in Turkey [21], the questions of early menarche, late menopause, and family history were answered correctly by 48.7%, 53.5% and 87.9% of the students, respectively. In general, regardless of the country where the research was conducted, medical school students were more likely to know breast cancer risk factors than other university students. The results of our study were similar to studies conducted with university students in other countries.

Modifiable lifestyle risk factors are important to reduce and prevent breast cancer risks [8]. In our study, students knew about lifestyle factors (such as low physical activity, obesity, smoking and alcohol use, and stress) at a higher rate than other risk factors. This may be because students in our study were generally aware of the importance of lifestyle changes that are effective in preventing many chronic diseases [34]. In other studies, lifestyle risk factors were less known as breast cancer risk factors compared to our study [13, 25, 35, 36].

In our study and other studies, students’ knowledge of breast cancer symptoms was higher than the risk of breast cancer [11, 13, 37]. This may be because breast cancer symptoms are seen more frequently on social media [38], or because students expect breast-related changes in breast cancer to occur, so the rate of correct answers may have increased.

In our study, students learned about breast cancer mostly from social media (29.5%) and medical websites (27%). The rate of learning from pharmacists was quite low (5.9%). Pharmacists have an important role in patient counseling and increasing patient awareness about breast cancer. Pharmacists can educate patients about BSE, CBE, mammograms and provide lifestyle advice, and produce educational brochures about breast cancer risk factors and symptoms.They can also inform patients about the side effects of the anticancer drugs they use and possible drug-drug interactions with other medications [23, 39]. In this context, pharmacists need to update their knowledge through in-service training and take a more active role in patient counseling. In studies conducted in various countries, students’ sources of information varied, but social media was more prevalent. In a study in Saudi Arabia [40], students’ sources of information were awareness campaigns (54.1%), internet (38.6%) and television (38.6%). In a study conducted in Egypt [33], social media was the source of information for most students (42.9%). Unlike the study conducted on medical faculty students in Turkey and Syria [11, 21], the students’ source of information was courses (39.2% and 70.4%, respectively). This may be because more emphasis is given to courses related to cancer awareness in medical school or that students are less interested in social media because they have very busy course schedules.

In some studies, it was found that the academic year affected the level of breast cancer knowledge (*p* < 0.05) [25, 29]. In the study of Ismail et al. [11], it was found that grade point average, maternal education level, smoking, and alcohol use affected the level of breast cancer knowledge (*p* < 0.05). In our study, no statistical significance was found in any of the variables related to breast cancer risk factors and symptoms (*p* > 0.05). The reason why there is no significant difference between the academic year and breast cancer knowledge level may be that breast cancer is not mentioned in any of the courses at the faculty. To eliminate educational gaps, it is necessary to include courses on breast cancer awareness in the faculty. It is also clear that different educational interventions such as social media, television, and brochure distribution are needed.

### Strengths and limitations of the study

To the best of our knowledge, this is the first study investigating the breast cancer knowledge level of pharmacy students in Turkey. Additionally, the rate of students who were interested in the research was not bad (74.5%).

One of the limitations of our study was that it could not be generalized nationally because it was conducted in a single faculty.

### Recommendations

We recommend conducting multicenter, large-scale studies. Additionally, studies can be conducted in the future to investigate the effects of various educational interventions on students’ knowledge levels and to compare the effects of educational interventions. For this purpose, controlled studies or pre-post intervention studies can be conducted. It would also be useful to investigate the long-term effects of these educational interventions.

## Conclusion

This study showed that students’ knowledge of breast cancer risk factors was poor, but their knowledge of breast cancer symptoms was acceptable. Additionally, no statistical significance was found in any of the variables related to breast cancer risk factors and symptoms in this study. Since determining breast cancer risk factors and symptoms is important in preventing breast cancer, students’ deficiencies in this regard should be eliminated. For this reason, breast cancer awareness courses should be included in faculties. Different educational interventions such as social media, television, and brochure distribution need to be further included. In addition, the Ministry of Health needs to give more importance to regular training at universities on breast cancer awareness and early detection.

## Data Availability

The datasets used and/or analysed during the current study available from the corresponding author on reasonable request.
